# High‐Temperature Quantum Tunneling and Hydrogen Bonding Rearrangements Characterize the Solid‐Solid Phase Transitions in a Phosphonium‐Based Protic Ionic Liquid

**DOI:** 10.1002/chem.202200257

**Published:** 2022-03-28

**Authors:** Alexander E. Khudozhitkov, Masaki Donoshita, Alexander G. Stepanov, Frederik Philippi, Daniel Rauber, Rolf Hempelmann, Hiroshi Kitagawa, Daniil I. Kolokolov, Ralf Ludwig

**Affiliations:** ^1^ Boreskov Institute of Catalysis Siberian Branch of Russian Academy of Sciences Prospekt Akademika Lavrentieva 5 Novosibirsk 630090 Russia; ^2^ Novosibirsk State University Pirogova Street 2 Novosibirsk 630090 Russia; ^3^ Division of Chemistry Graduate School of Science Kyoto University Kitashirakawa-Oiwakecho, Sakyo-ku Kyoto 606-8502 Japan; ^4^ Physikalische Chemie Universität des Saarlandes Campus B2.2 66123 Saarbrücken Germany; ^5^ Department LL&M University of Rostock Albert-Einstein-Str. 25 18059 Rostock Germany; ^6^ Institut für Chemie Abteilung für Physikalische Chemie Universität Rostock Dr.-Lorenz-Weg 2 18059 Rostock Germany; ^7^ Leibniz-Institut für Katalyse Universität Rostock e.V. Albert-Einstein-Str. 29a 18059 Rostock Germany

**Keywords:** ion pairing, ionic liquids, molecular mobility, quantum tunnelling, solid state NMR

## Abstract

We report the complex phase behavior of the glass forming protic ionic liquid (PIL) d3‐octylphosphonium bis(trifluoromethylsulfonyl)imide [C_8_H_17_PD_3_][NTf_2_] by means of solid‐state NMR spectroscopy. Combined line shape and spin relaxation studies of the deuterons in the PD_3_ group of the octylphosphonium cation allow to map and correlate the phase behavior for a broad temperature range from 71 K to 343 K. In the solid PIL at 71 K, we observed a static state, characterized by the first deuteron quadrupole coupling constant reported for PD_3_ deuterons. A transition enthalpy of about 12 kJ mol^−1^ from the static to the mobile state with increasing temperature suggests the breaking of a weak, charge‐enhanced hydrogen bond between cation and anion. The highly mobile phase above 100 K exhibits an almost disappearing activation barrier, strongly indicating quantum tunneling. Thus, we provide first evidence of tunneling driven mobility of the hydrogen bonded P−D moieties in the glassy state of PILs, already at surprisingly high temperatures up to 200 K. Above 250 K, the mobile phase turns from anisotropic to isotropic motion, and indicates strong internal rotation of the PD_3_ group. The analyzed line shapes and spin relaxation times allow us to link the structural and dynamical behavior at molecular level with the phase behavior beyond the DSC traces.

## Graphical abstract

**Tunneling driven dynamics** of the d3‐octyl phosphonium cation in an ionic liquid is observed by means of solid‐state NMR spectroscopy. Deuteron line‐shape analysis and relaxation time measurements provide information about the structure, mobility and rearrangements of the cations for the broad temperature range between 73 and 343 K. Differently strong hydrogen bonds are reported for the solid, supercooled and liquid state of the IL and map the change in molecular ordering upon phase transformation.

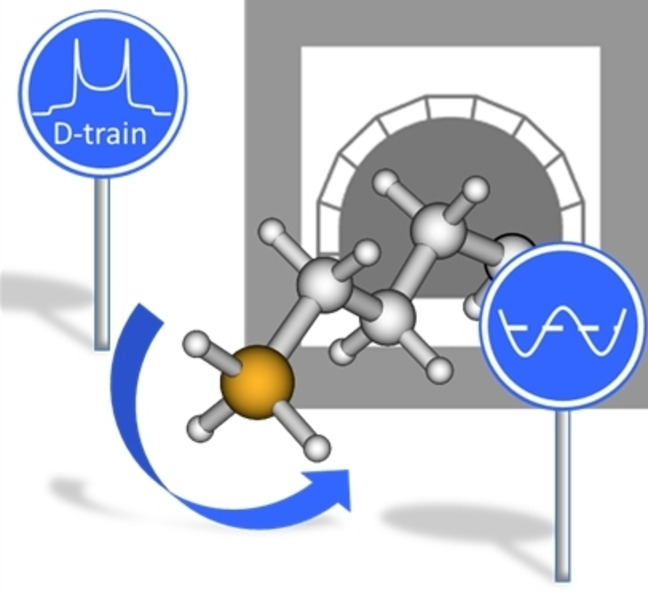

## Introduction

Ionic liquids (ILs) attract increasing interest in science and technology due to their unique properties, which can be tailored for specific applications ranging from chemical synthesis and separation processes to media for electrochemical devices.^[1]^ It is evident that a better understanding of their behavior at the microscopic scale will help to elucidate macroscopic fluid phenomena, and thus promote industrial applications. The favorable properties of these innovative fluids result from the delicate balance of Coulomb interactions, hydrogen bonding and dispersion forces.^[2]^ Designing these properties requires fundamental understanding of the strength, locality and directionality of the different types of interactions and how they contribute to the overall phase behavior.^[3]^ In particular, local and directional hydrogen bonding has significant influence on the structure and dynamics of ILs.[^3a^, ^3b^] Structure‐property relationships have long guided the discovery and optimization of novel materials and provide new insights into the nature of these unique materials.^[4]^ The class of protic ionic liquids (PILs) is of particular interest, because PILs form charge‐assisted hydrogen bonds between cations and anions. The specific nature of hydrogen bonding provides new dimensions for designing physicochemical properties such as melting temperature, viscosity and conductivity.^[1]^ Recently, we were able to show that hydrogen bonds between cation and anion, but also between ions of like charge, control the solidification route and lead either to a crystalline solid or to a glass.^[5]^ Although essential, the molecular mechanism of the complex phase behavior is still poorly understood and characterized.

**Figure 1:**
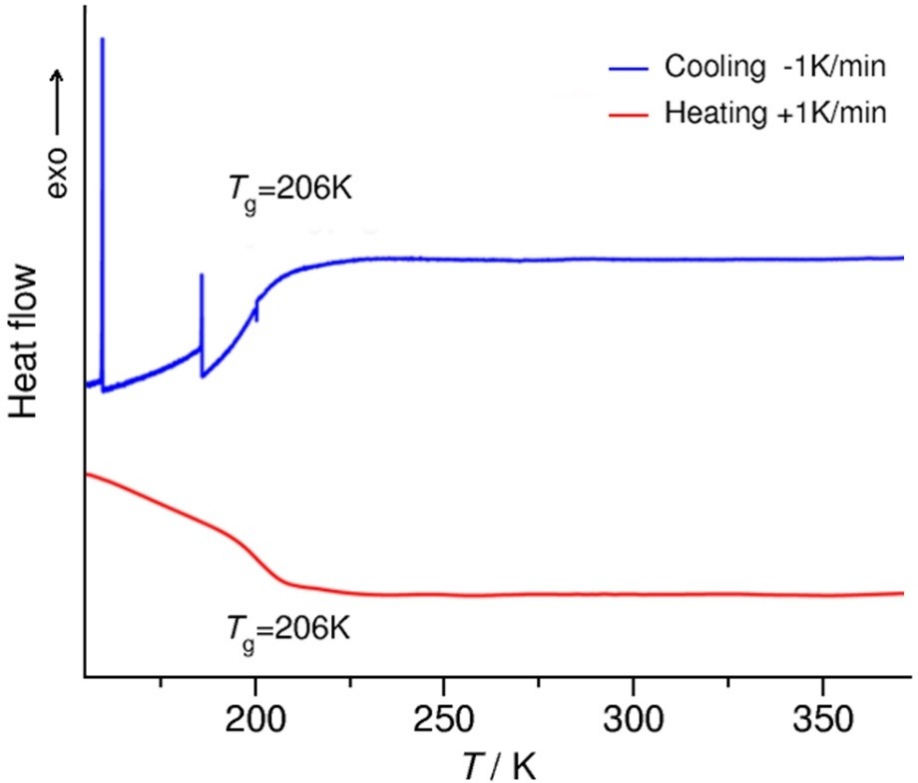
DSC thermograms of the deuterated PIL [C_8_H_17_PD_3_][NTf_2_] with indicated transition temperatures. Exothermal transitions are plotted as positive peaks.

For this reason, we have chosen the PIL d3‐octyl phosphonium bis (trifluoromethylsulfonyl) imide [C_8_H_17_ **PD_3_**][NTf_2_] as suitable model compound. Compared to alkylammonium PILs, the phosphonium‐based PILs have only weak hydrogen bonds. The flexible octyl group at the cation and the weakly interacting NTf_2_ anion allow investigations from the solid to the glassy and finally to the liquid state of this PIL covering a broad temperature range. We show that solid‐state ^2^H NMR spectroscopy allows studying the structure, hydrogen bond mobility and phase transition behavior in this type of PILs. The virtue of the ^2^H NMR compared to the more common ^1^H case is in the perfect labelling selectivity and the quadrupolar nature of the deuterium nuclei, providing thus of the spectroscopic data mapping the structure and dynamics of the deuterated species only. We combined line shape and spin relaxation studies of the deuterons in the PD_3_ group of the octylphosphonium cation for mapping and correlating the phase behaviour for a broad temperature range from 71 K to 343 K. Pake spectra of the static state at low temperatures should provide the first deuteron quadrupole coupling constants (DQCC) measured for P−D deuterons. The observed transition enthalpy from static to mobile states with increasing temperature reveals promising information about the breaking of weak, charge‐enhanced hydrogen bonds between cation and anion. We put particular focus on the highly mobile phase above 100 K exhibiting an almost disappearing activation barrier, a good indicator for quantum tunnelling. Thus, we could provide the first evidence of tunnelling driven mobility of the hydrogen bonded P−D moieties in the glassy state of a PIL, at surprisingly high temperatures of about 200 K. Above 250 K, the mobile phase turns from anisotropic to isotropic motion, and indicates strong internal rotation of the PD_3_ group. The analysed line shapes and spin relaxation times are reflected in the DSC traces and allow drawing conclusions for the structural and dynamical behaviour at molecular level (see Figure 1).

## Results and Discussion

### First deuteron quadrupole coupling constant for PD_3_ from spectra in the deep frost

**Figure 2:**
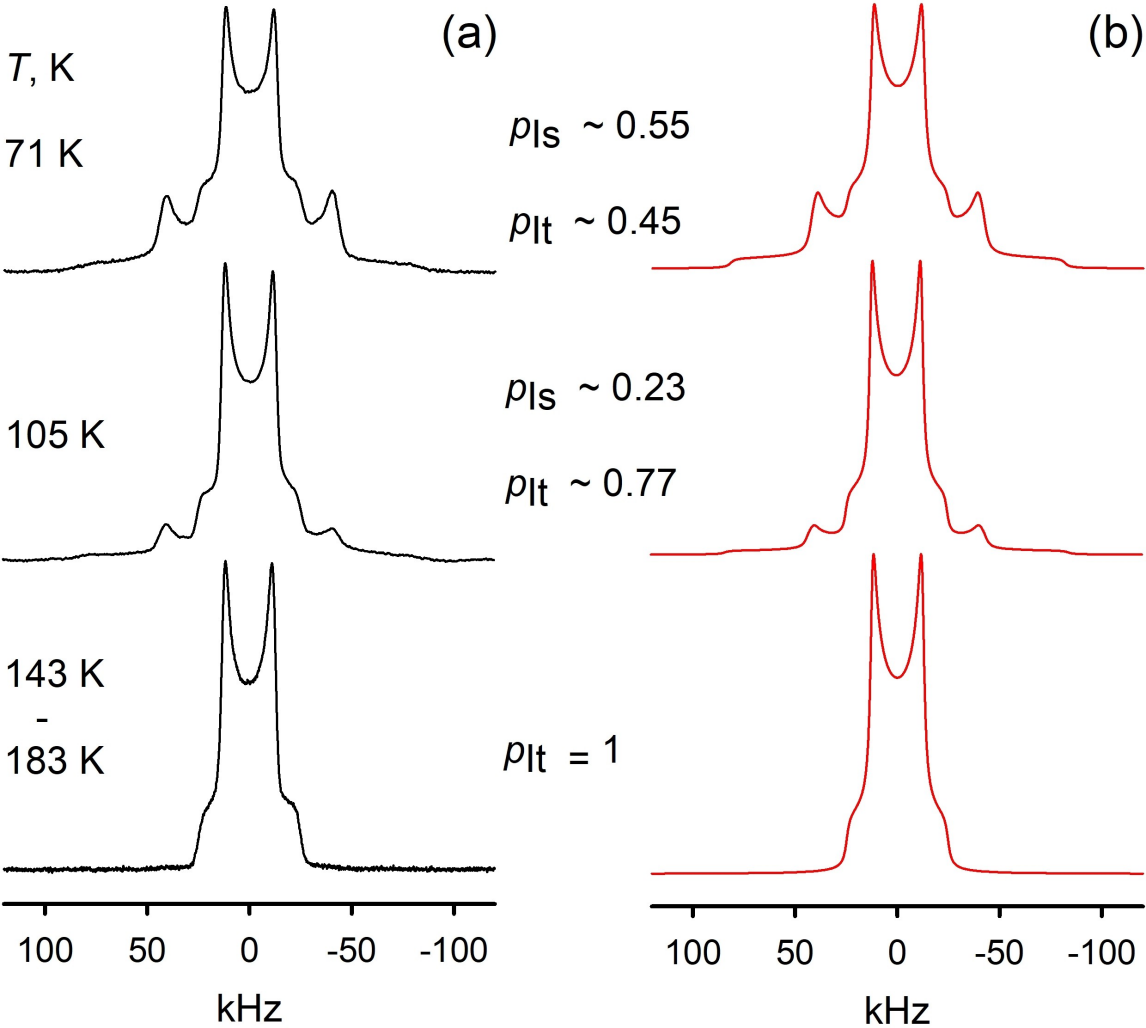
^2^H NMR spectra line shape temperature evolution of the PIL [C_8_H_17_PD_3_][NTf_2_]: a) experimental; b) simulated. See further in Supporting Information Figure S1. The experimental patterns shown on Figure 2 are composed by two signals, a broad one *I*_s_ with (*Q*_Is_=111 kHz, *η*_Is_=0.04) and narrow one state *I*_t_ with (*Q*_It_=33 kHz, *η*_It_=0.0), both anisotropic with a typical Pake‐powder line shape. Spectra deconvolution is given on Figures 3 and S1.

The ^2^H solid‐state NMR spectra are characterized by the deuteron quadrupole coupling constant DQCC, *χ*_D_=(*e^2^q_zz_Q/h*), and the asymmetry parameter, *η*=(*q_xx_*−*q_yy_)*/*q_zz_*.^[6]^ The DQCC describes the coupling between the nuclear quadrupole moment *Q* and the main component of the electric field gradient (*EFG*) tensor, *q_zz_*. It reflects the electronic environment and is thus a sensitive probe for hydrogen bonding. The asymmetry parameter *η* is characterized by the principle components *q_xx_*, *q_yy_* and *q_zz_* of the electric field gradient tensor and provides information about the shape of the electric field gradient.^[7]^ If the experimental line shape can be deconvoluted into multiple signals with individual quadrupole coupling parameters then the sample contains different types of hydrogen bonds. Here, we determined the first DQCC from the solid‐state deuterium NMR powder patterns of the PIL in temperature ranges between 71–183 K. Covering such a broad temperature range beyond the glass transition provides deeper understanding of hydrogen bonding and molecular ordering in glassy PILs. In Figure 2, we show the ^2^H NMR spectra for the PIL obtained at 71 K, 105 K, and throughout for the temperature range between 143 and 183 K. All spectra show two Pake patterns providing two different deuteron quadrupole coupling constants, *χ*_D_=$Q_{Is}$ and *χ*_D_=$Q_{It}$ .

Both spectral parameters are sensitive enough to distinguish different hydrogen bonding states as shown for intermolecular cation‐anion and cation‐cation hydrogen bonding, where the latter is characterized by hydrogen bonds between the functional groups of the like‐charged ions.^[8]^

**Figure 3:**
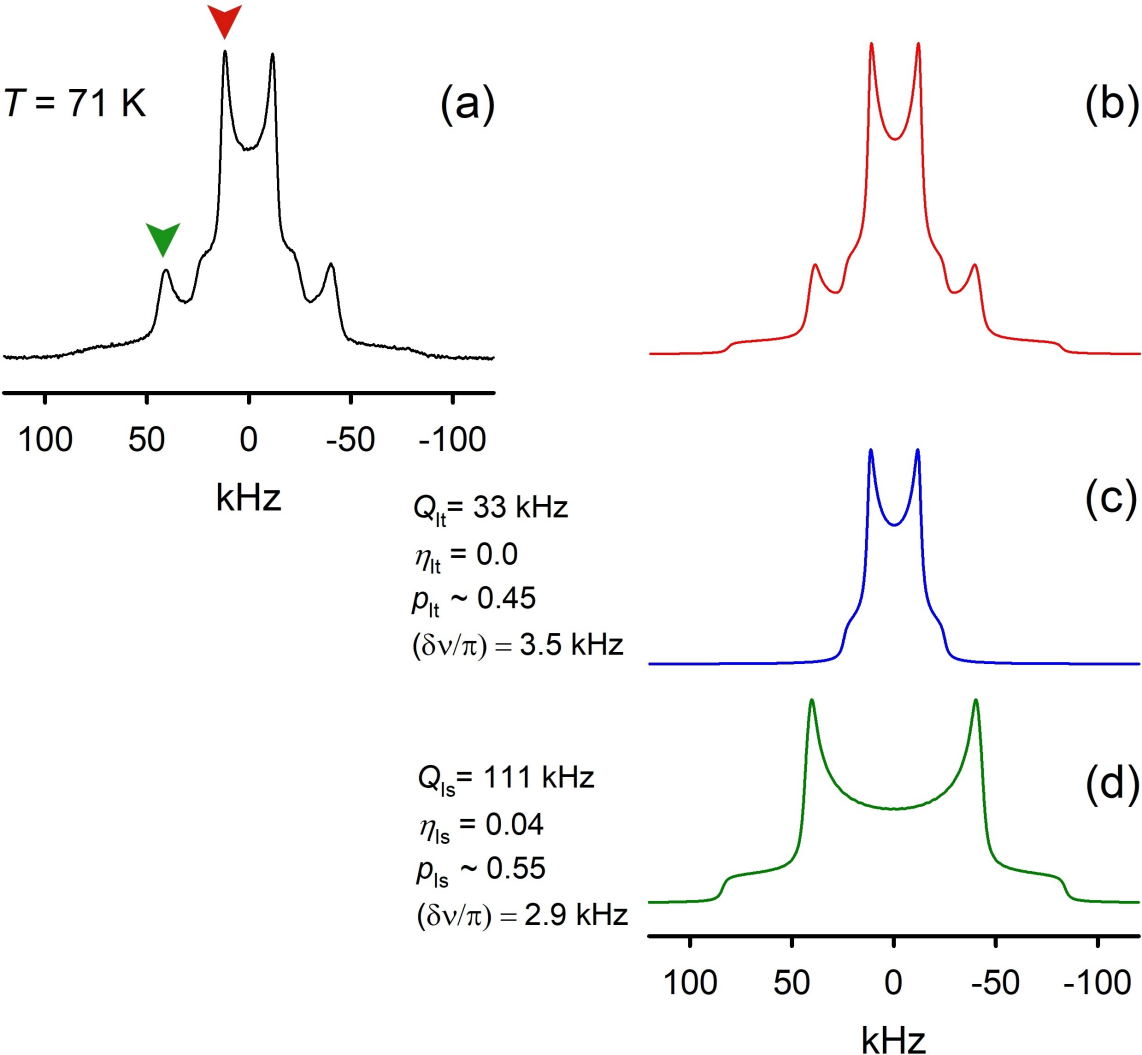
^2^H NMR spectra deconvolution for PIL [C_8_H_17_PD_3_][NTf_2_] at T=71 K: a) experimental; b) simulated; simulation deconvolution on signals c) from the *I*_t_ and d) *I*_s_ components.

**Figure 4:**
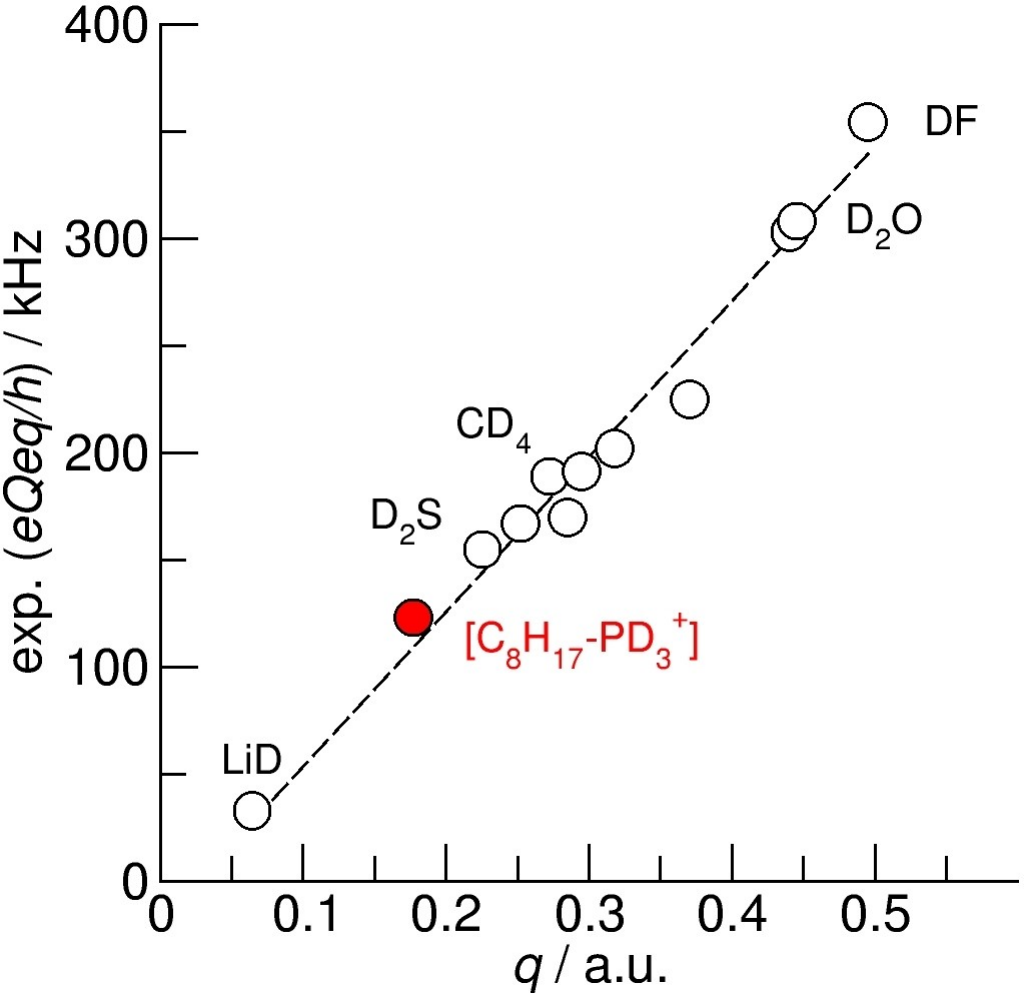
Experimental quadrupole coupling constants (*eQeq/h*) plotted versus B3LYP‐D3/6‐31+G* calculated electric‐field gradients *q* for deuteron nuclei in small molecules such as DF, D_2_O, CD_4_, D_2_S or LiD etc. as described by Huber.[21,22] The slope gives a reasonable NQM (namely, 295.5 fm^2^) for this level of theory and can be used for calculating deuteron quadrupole coupling constants for the deuterons in PIL cation [C_8_H_17_PD_3_^+^].

**Figure 5:**
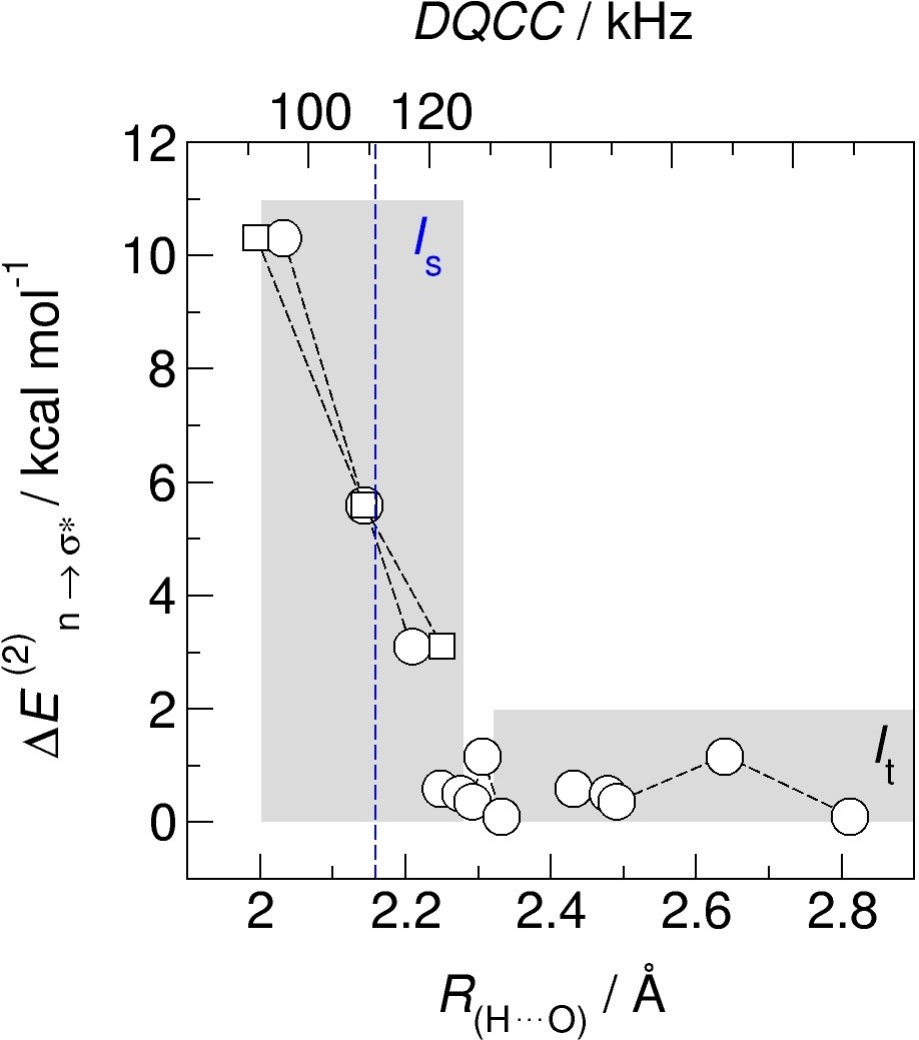
NBO calculated second order stabilization energies Δ*E*(2)_n_→_σ*_ (open circles) plotted versus calculated intermolecular hydrogen bond distances, *R*_(H⋅⋅⋅O)_ (below) and calculated DQCC for monomer and dimer ion pairs of [C_8_H_17_PD_3_][NTf_2_]. Reasonable stabilization energies are only obtained for distances *R*_(H⋅⋅⋅O)_ between 2.03 and 2.21 Å (circles) related to DQCCs between 91.3 and 122.1 kHz (squares). We consider the static species *I*_**s**_ species as weakly hydrogen bonded described by the measured DQCC of 111 kHz (dashed vertical blue line). Non‐hydrogen bonded species with *R*_(H⋅⋅⋅O)_>2.3 Å belong to the mobile fraction *I*_t_.

The *q_zz_* as the highest EFG gradient value is aligned to the P−D bond and mainly follows the hydrogen bond direction, hence for a given deuteron (proton) species, the line shape change is determined by bonding properties as well as rate and geometry of its molecular reorientations. In order to get the static non‐averaged DQCC (commonly denoted as *Q*_0_) we must inspect the measured spectra at the lowest accessible temperature. However, even at 71 K the pattern still shows the presence of two signals, representing the static *I*_s_ and the mobile *I*_t_ fractions, respectively. Above 143 K, only the narrow component *I*_t_ remains. The shapes of the two components do not change upon cooling hence we might conclude that the broad component *I*_s_ represents the fully immobilized, static deuterons in the PD_3_ fragment. In such case the narrow signal *I*_t_ represents the result of the deuterons dynamics. The *I*_t_ effective DQCC is approximately three times smaller compared to the *I*_s_ case and shows no significant asymmetry, which hints that the motion responsible for this narrowing is an axially symmetric anisotropic rotation. Numerical analysis then follows the procedures proposed by Abragam^[6e]^ and developed in more detail by Spiess, Wittebort and Kolokolov.^[9]^ The fitted spectra are obtained by Fourier transform of the powder‐average taken over the polar angles *θ* and *ϕ* of the correlation function *G(t,θ,ϕ)*, which governs the time evolution of the transverse ^2^H spin magnetization after the solid echo pulse sequence (see Figure 3). Applying such a scheme and taking *Q*_PD_=*Q*_IS_=111 kHz, the resulting rotational angle *θ*_PD_=110.5°, leading to the narrow spectrum component with *Q*_IT_=33 kHz, perfectly corresponds to the known C‐PD_3_ fragment geometry. The rotation rate at all temperature is found in the fast exchange limit on the ^2^H NMR time scale, that is, *k*_C3_ ≫ *Q*_PD_ ∼10^5^ Hz. Hence, we report the first experimental DQCC of the hydrogen bonded P‐D species in solids and a first example of the anisotropic rotation of these hydrogen bonds in solid PILs. How reliable is the measured DQCC value of about 111 kHz in the solid state? To the best of our knowledge, no gas phase values for the DQCC of P−D bonds from microwave studies are available, probably because it is highly toxic respiratory poison. Similar may be true for other phosphines. Thus, we calculated all components of the electric field gradient tensor at the B3LYP−D3/6‐31+G* level of theory for PD_3_, PD_4_^+^ and C_8_H_17_PD_3_^+^ monomeric species.^[10]^ Because the calculated *EFG* at the nuclei are method and basis set dependent, we need to calibrate nuclear quadrupole moments (NQM). For that purpose, we plotted the measured gas phase DQCCs from microwave spectroscopy versus calculated electric field gradients for small molecules such as CD_4_, CD_3_OH, HNCO etc. as described by Huber et al.[^7a^, ^7b^] The slope gives a reasonable NQM value (namely, 295.5 fm^2^), which can be then used for calculating accurate DQCCs for monomer or gas phase species at the given DFT‐level (see Figure 4). Using this approach, we obtained DQCC=123 kHz for the gas phase monomer [C_8_H_17_PD_3_^+^], about 10 % above the measured solid‐state value of 111 kHz. Of course, any specific interaction with the environment should result in shorter intermolecular hydrogen bond distances, *R*_(H⋅⋅⋅O)_ and smaller DQCCs. The strength of hydrogen bonding can be readily recognized in the framework of the natural bond orbital (NBO) analysis as distinctive n_O_→σ*_PH_ donor‐acceptor interactions expressed by the second order stabilization energies Δ*E* ^(2)^_n→σ*_.^[11]^ Charge from the oxygen lone pair orbitals of the [NTf_2_]^−^ anion is donated into the anti−bonding orbital of the PH group. Because NBO−based charge transfer descriptors demonstrate strong correlative relationships with known experimental signatures of hydrogen bonding, we plotted Δ*E*(2)_n_→_σ*_ versus intermolecular distances *R*_(H⋅⋅⋅O)_ and DQCC for monomer and dimer ion pairs of [C_8_H_17_PD_3_][NTf_2_]. We principally observed two states for the deuterons in the PD_3_ groups, which we refer later on to a static state *I*_s_ and a mobile state *I*_t_. As shown in Figure 5, reasonable stabilization energies are only obtained for distances *R*_(H⋅⋅⋅O)_ between 2.03 and 2.21 Å related to DQCCs between 91.3 and 122.1 kHz, respectively. We refer these weakly hydrogen bonded species to the static state *I*_s_, well described by the measured DQCC of 111 kHz (dashed vertical blue line in Figure 5). The non‐hydrogen bonded species exhibiting no specific interaction were obtained for *R*_(H⋅⋅⋅O)_>2.3 Å. In this state, the calculated DQCCs are similar to the gas phase value and the second order stabilization energy almost vanishes. Hydrogen bonding leads to shortening of the intermolecular distance *R*_(H…O)_ of about 0.4 Å and lengthening of the intra molecular bond PH of about 0.01 Å. Both, the intra and intermolecular geometry changes decrease the DQCC down to 91 kHz. A crude comparison supports the result and conclusion. In water, the OH bond distance is lengthened from 94.5 pm in the gas phase to 101 pm in hexagonal ice.^[12]^ The corresponding DQCCs are 318 kHz and 220 kHz, respectively.^[13]^ Thus, an increase of 1 pm in OH bond length corresponds to a decrease of 15 kHz in the coupling parameter, almost similar to our case. In our calculation we obtain an average value DQCC=108 kHz which is close to the experimentally observed value of about 111 kHz in the static environment at low temperatures.

### Population of static and mobile fractions

**Figure 6:**
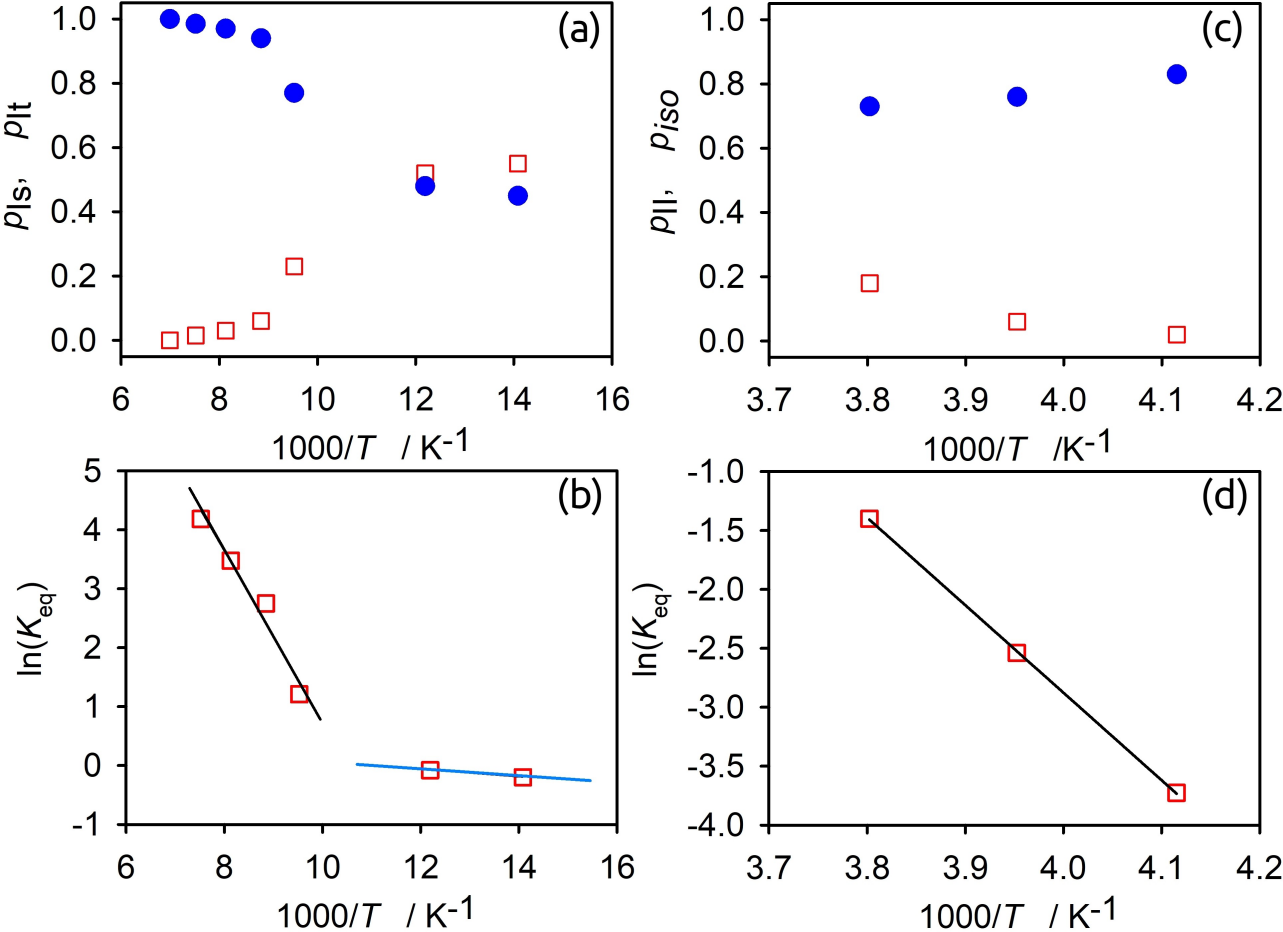
Temperature evolution analysis for [C_8_H_17_PD_3_][NTf_2_] (a‐b) at low temperature states *I*_t_ and the static state *I*_s_: a) relative populations for static *I*_s_ (□) and mobile *I*_t_ (•) states; b) van't Hoff plot for the equilibrium constants *K*_eq_=*p*_It_/*p*_Is_. c and d) At high temperature states *II* and the isotropic state iso: c) relative populations for mobile iso (□) and anisotropic iso (•) states; d) van ‘t Hoff plot for the equilibrium constants *K*_eq_=*p*_iso_/*p*_II_.

The fact that the ‐PD_3_ rotation is always fast even at 71 K, indicates, that the ratio between the static *I*_s_ and mobile *I*_t_ spectral components is governed only by the population factor. Such occurrence usually indicates on a very low activation barrier for the motion which happens when the internal rotations at low temperature are governed by tunneling rather than a common activation process.[^12^, ^13^] In such a case, the quantitative analysis of the relative populations of each state provides additional information on the thermodynamics of the transition to the mobile state, via the van't Hoff equation,

(1) $$ln\left(K_{eq}\right)=-\frac{\Delta H^{\theta }}{RT}+\frac{\Delta S^{\theta }}{R},$$

where the equilibrium constant is obtained from the ratio between relative populations of the mobile state *I*_t_ and the static state *I*_s_, i. e., $K_{eq}=p_{It}/p_{Is},$ as a function of the temperature (see Figure 6a) and b). The van't Hoff plot yields two distinct molar enthalpy change *ΔH* ^Θ^ for the transition to mobile state (Δ*H* ^*Θ*^=0.53 kJ mol^−1^, *ΔS* ^*Θ*^=5.7 J mol^−1^ K^−1^) and (Δ*H* ^*Θ*^=12 kJ mol^−1^, Δ*S* ^*Θ*^=125 J mol^−1^ K^−1^). This, somewhat unexpected at the first glance result holds important information about the cation‐anion arrangement in the solid PIL, i. e., that within the solid phase the ‐PD_3_ fragment of the cation has at least two distinct surroundings, two possible localizations within cation‐anions clusters and that the population factors of these two positions are comparable.

What does this information contribute to the physical picture of the frozen PIL? Strong directional interactions, like strong hydrogen bonds are widely accepted to be blocking the tunneling‐driven dynamics.^[14]^ Thus, the ‐PD_3_ fragments present in our system are either weakly hydrogen‐bonded or non‐hydrogen‐bonded at all (see Supporting Information). For a tunneling‐driven rotation, we have two possible scenarios: a) the whole population of rotating species is simultaneously involved into the process, with a rate slowly (non‐exponentially) increasing with the temperature change, b) the rotor represents a typical 2‐level system, where in the ground state only restricted librations are possible while in the excited state the tunneling mode is fast and almost temperature independent. In the latter case, the relative populations are governed by the energy needed to jump from the ground level to the excited one, i. e., by the transition enthalpy. This scenario can only be observed when the rate of the exchange between the states is slow. The present case clearly falls into the second scenario. Having two distinct transition enthalpies simply indicates two different interaction strengths of the P−D deuterons with the local molecular environment. Keeping in mind that strong interaction hinders the tunneling completely, we can conclude, that in the case of the low transition enthalpy the ‐PD_3_ deuterons are nearly free from any interaction with the anions, while for the second population a certain interaction is present, and the P−D⋅⋅⋅O hydrogen bond can be expected to be shortened compared to the first case. Still, we need to note that since the only one DQCC is actually observed the potential hydrogen bond in the second case must be very weak.

### Tunneling‐driven relaxation processes

**Figure 7:**
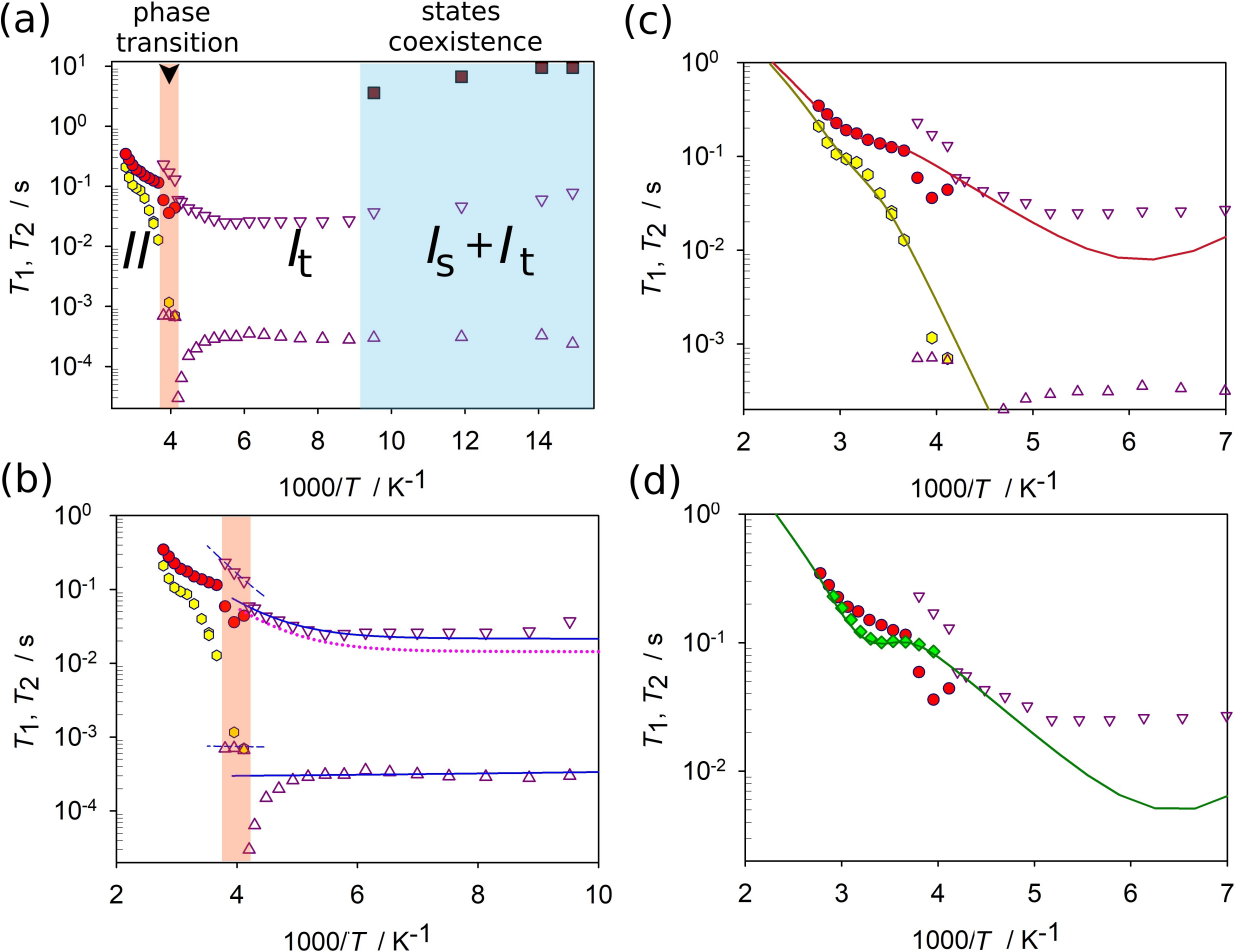
^2^H NMR spin relaxation temperature dependences: a–c) experimental *T*_1_ (▿) and *T*_2_ (▵) for anisotropic mobile state *I*_t_; experimental *T*_1_ (▪) for the anisotropic static state *I*_s_; experimental *T*_1_ (•) and *T*_2_ (◊) for isotropic mobile state II; simulations are given in solid lines for stable phases and in dashed lines for the transition region (marked in red); dotted lines show the *T*_2_ simulation results for a simplified model where only a single, fast uniaxial rotation is considered. d) Experimental *T*_1_ (♦) for the isotropic mobile state II measured at 38.38 MHz resonance frequency; simulation is given by a solid line. All other experimental data was measured at 61.4 MHz resonance frequency.

In the absence of the line shape change the kinetics of the hydrogen bond rotation can be accessed by the spin‐relaxation analysis. Since for an anisotropic pattern the relaxation times are also in general anisotropic, the measurements and the numerical analysis were usually performed at a specific spectral position (arrow‐marked on Figure 3) corresponding to a polar observation angle *θ*=90^○^.[^9b^, ^9c^, ^15^] The experimental results for state *I*_t_^2^H NMR *T*_1_ and *T*_2_ relaxations temperature dependences along with their numerical fit are given on Figure 7(a) and (b).

**Figure 8:**
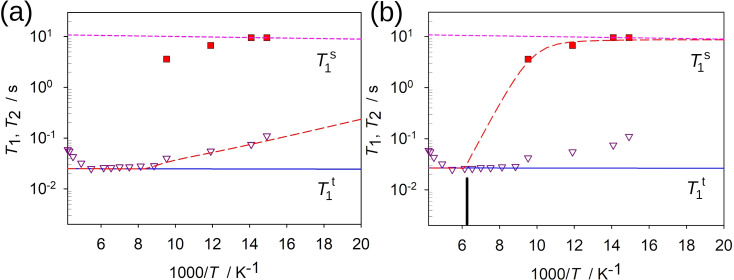
a and b) ^2^H NMR *T*_1_ spin‐lattice relaxation temperature dependences for the anisotropic static *I*_s_ and mobile *I*_t_ states: experimental *I*_t_−*T*_1_ (▿) for *I*_s_−*T*_1_ (▪). The blue solid line represents individual relaxation time *T*_1_^t^ for the *I*_t_ state, the pink short‐dashed line for the individual relaxation time *T*_1_^s^ for the *I*_s_ state, the exchange results is given by the red long‐dashed line. The exchange process is governed by the equilibrium constants derived from the line shape analysis: in a) the fit is based on the ${K_{eq}}^{1}$ with small transition enthalpy, in b) with the ${K_{eq}}^{2}$ with the larger one. The exchange *rate* constant does not exceed 10 Hz in each case, perfectly in accordance with line shape analysis. The individual relaxation times are fitted using the following model: for *I*_s_ the P−D is assumed exhibit barrierless restricted librations with angular amplitude of <5° and rate $\frac{{k_{lib}}^{s}}{2\pi }$ =1×10^10^ Hz, for *I*_s_ the P−D is assumed to exhibit uniaxial *C*_3_ rotation within the ‐PD_3_ geometry with ${k_{C3}}^{t}$ (*E* ^t^=0.1 kJ mol^−1^; $\frac{{k_{C30}}^{t}}{2\pi }$ =5×10^9^ Hz). The black line in b) shows the approximated point where by the exchange model predicts full transition to the dynamical state.

From 71 K to 113 K *T*_1_ relaxation shows very slow decrease, which is associated with very slow (≪10^3^ Hz) exchange between different dynamical states *I*_s_ and *I*_t_: the *T*_1_ relaxation for static deuterons populating the *I*_s_ ∼10 times longer and it is possible to see its influence on the mobile species. However, above 113 K the *I*_s_ population becomes visually negligible and thus the measured relaxation represents the pure mobile state *I*_t_. The fact that the remaining *I*_s_ species keep having an independent from *I*_t_ relaxation time up to 113 K is fully consistent with the present of two distinct ‐PD_3_ species which are characterized by notably different transition enthalpies. A simple simulation model with two relaxation times (*T*_1_^s^, *T*_1_^t^) involved into a chemical exchange with two consecutive processes governed by the equilibrium constants derived from the line shape analysis perfectly depict the experimental observation (see Figure 8). Noteworthy, calculations predict that the second dynamical transition should be fully fulfilled at temperature ∼160 K, which perfectly coincides with the low transition observed by the DSC measurements (*T*_s2_ ∼158 K).

The temperature dependence of the *I*_t_ state relaxation from 113 K and up to *T* ∼200 K is flat, showing no notable slope. This is clear indication on tunneling‐driven relaxation process, i. e., when the P−D reorientation is fast with a close to zero activation barrier. Above 200 K the curve bends and relaxation starts to grow with a stable slope, which indicates that the motion passes to a more common activation‐driven process.^[4c]^ In the same time the *T*_2_ relaxation shows almost no change up to circa 220 K but is found notably smaller than *T*_1_. To the best of our knowledge this is the very first observation of the high‐temperature tunneling occurring in solid PILs. Recently, Do et al. reported quantum tunneling in the methyl groups of the crystallized ionic liquid [DMIm][TFSI] probed by quasi‐elastic neutron scattering (QE) experiments.^[16]^ However, methyl groups are not affected by Coulomb interaction or hydrogen bonding. Thus, it is not surprising that methyl tunneling is observed ILs as it has been detected in molecular systems.[^14a^, ^17^] Moreover, Do et al. could discuss tunneling phenomena only at very low temperatures (33 K) and facilitated by LiTFSI (LiNTf_2_) salt.^[16]^

At this stage, the quantitative analysis of these unusual curves could yield the motional model into play. As mentioned above, the spin relaxation times *T*_1_ and *T*_2_ are generally anisotropic and depend on the observation angles *θ* and *ϕ* in the powder pattern. They can be computed within a jump‐exchange model, assuming usually, that the jump exchange is a random Markovian process.^[15]^ With this in mind, we can pass to the numerical analysis of the state *I*_t_ anisotropic relaxation. We firstly apply the simplest model suggested by the line shape analysis: the dynamics is fitted by a uniaxial 3‐site jump‐rotation and thus we can use the same geometry, populations and an exchange matrix K. The only different is that the exchange matrix should reflect the tunneling and activation regimes for the rotation. This is realized straightforwardly by introducing two individual rotation rate constants: $k_{C3}=\;{k_{C3}}^{t}+{k_{C3}}^{A}$ , where index t stands for tunneling, and A for activation. Such a scheme perfectly reproduces the anisotropic *T*_1_ relaxation in state *I*_t_, but notably fails to reproduce the *T*_2_ relaxation, as shown on Figure 7(b). This actually shows, that in addition to the relatively fast uniaxial rotation, there exists a certain much slower process. Since the shape of the spectrum is stable at least up to 183 K, we must assume that this is some kind of highly restricted librational motion.

Such scheme gives a perfect fit of the experimental pattern within the region of interest. The drastic decrease of the *T*_2_ relaxation is associated with the increase of the slow librations amplitude discussed further. Here, we only recognize that this geometry change does not affect the much faster *T*_1_ relaxation.

The resulting Arrhenius parameters for uniaxial rotation ${k_{C3}}^{t}$ (*E* ^t^=0.1 kJ mol^−1^; $\frac{{k_{C30}}^{t}}{2\pi }$ =5×10^9^ Hz) and ${k_{C3}}^{A}\;$ (*E* ^A^=12 kJ mol^−1^; $\frac{{k_{C30}}^{A}}{2\pi }$ =3.8×10^11^ Hz), shows that we indeed have been able to characterize the almost‐barrierless tunneling‐driven rotation of the hydrogen bonded ‐PD_3_ fragment. The libration process is characterized by $k_{lib}$ (*E*_lib_=0.2 kJ mol^−1^; $\frac{k_{lib0}\;}{2\pi }$ =3×10^5^ Hz). Its slow rate indicates that this type of motion is not the individual libration of each P−D bond, but rather a slow libration of the whole ionic pair, which is typical for glassy states.^[18]^

**Figure 9:**
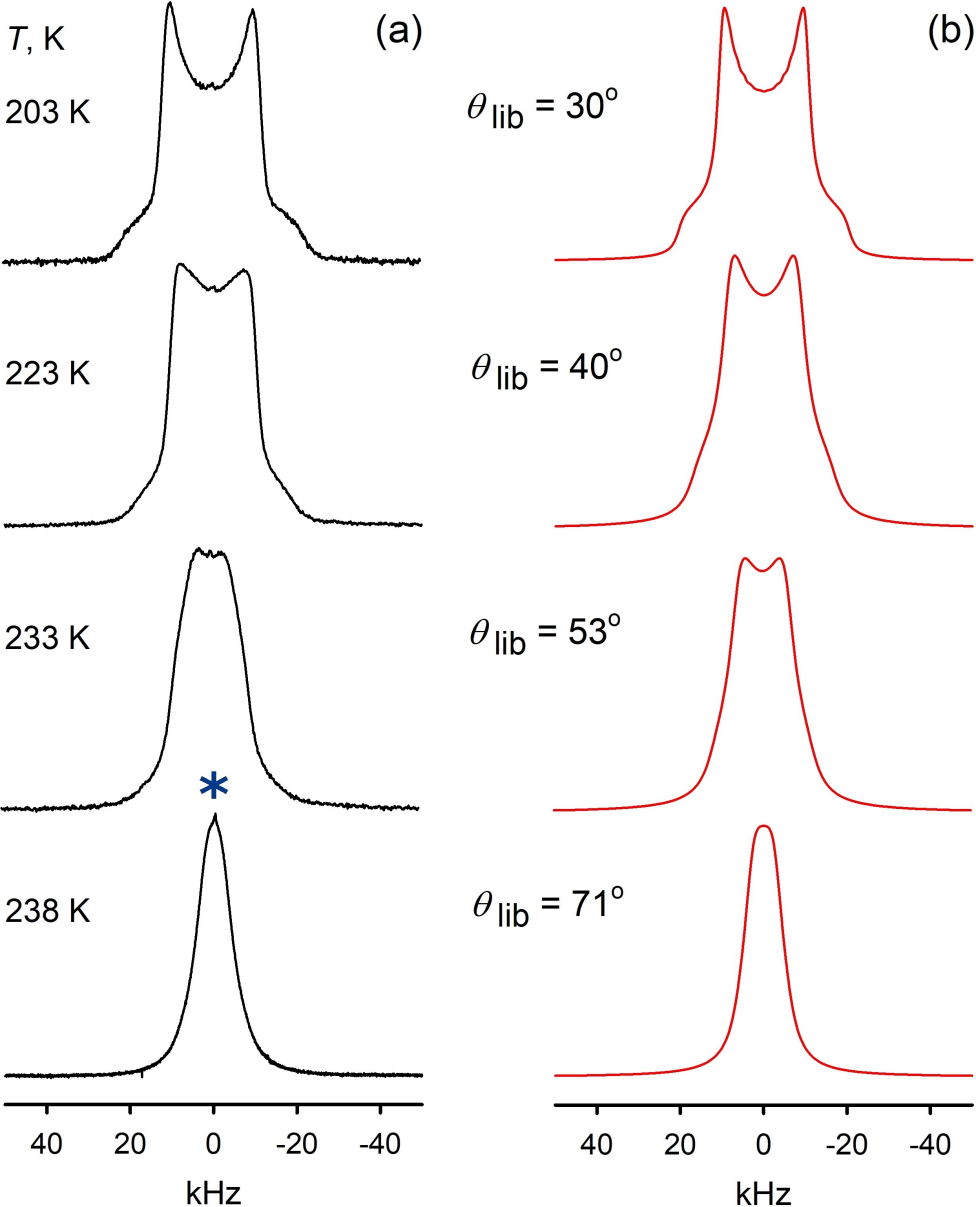
^2^H NMR spectra line shape temperature evolution of the [C_8_H_17_PD_3_][NTf_2_] PIL: a) experimental; b) simulated.

The substantial changes in the ^2^H NMR pattern starts above 203 K as shown in Figure 9. This temperature almost perfectly coincides with glass transition at *T*_g_=206 K observed in the DSC traces (Figure 1). The observed line shape evolution shows, that upon heating the pattern becomes broadened and eventually narrowed down to a seemingly isotropic shape. Numerical analysis shows, that such behavior is rationalized by librations in a cone motions^[9b]^ which progressively increase their amplitude up to almost realizing the full isotropic tumbling. Note that during simulation we have assumed that the libration rate is in a fast regime, which agrees with the $k_{lib}$ rate extracted from the relaxation analysis. The drastic change in the line shape in this temperature range perfectly correlates with the drop in anisotropic *T*_2_ relaxation discussed above.

**Figure 10:**
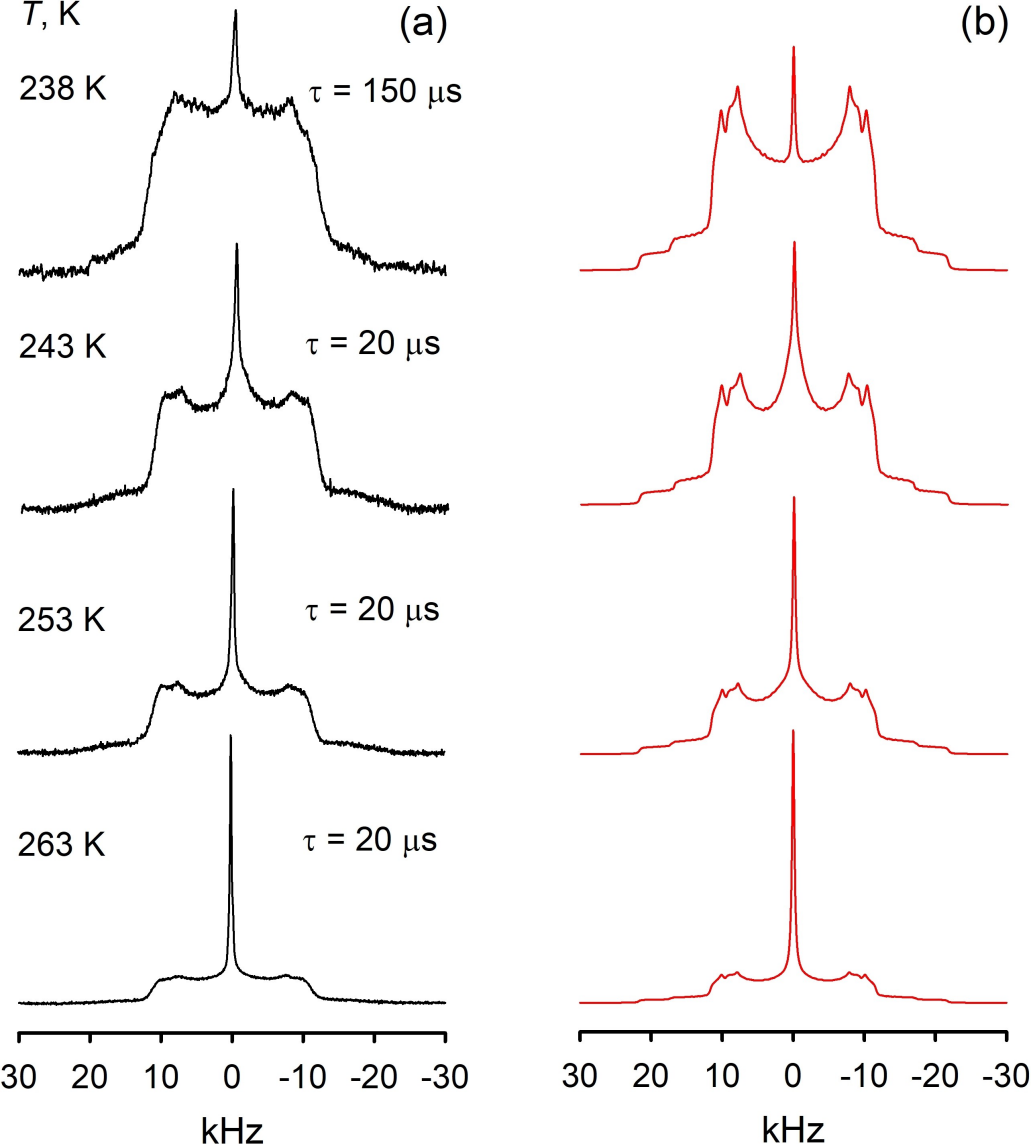
^2^H NMR spectra line shape temperature evolution of the [C_8_H_17_PD_3_][NTf_2_] PIL: a) experimental; b) simulated. See Figure S2 for deconvolution details.

At any rates, it seems obvious, that such an increase in the libration amplitude indicates some phase changes in the still solid PIL. Indeed, at 238 K the presence of a new phase becomes visible in the spectra line shape: due to the small population, the new phase in the patterns acquired by standard acquisition parameters with the refocusing delay *τ*=20 μs it is manifested by a subtly small isotropic peak (marked by asterisk on Figure 9). However, measuring the same patterns with larger delay *τ*=150 μs allows to filter out the low temperature phase *I*_t_ (the so‐called *T*_2_‐filtration) and picture clearly the pattern characterizing the new phase (Figures 10 and S2). Since we are already notably above glass‐transition temperatures at 206 K we could state that this new phase represents the supercooled liquid phase of the PIL.

**Figure 11:**
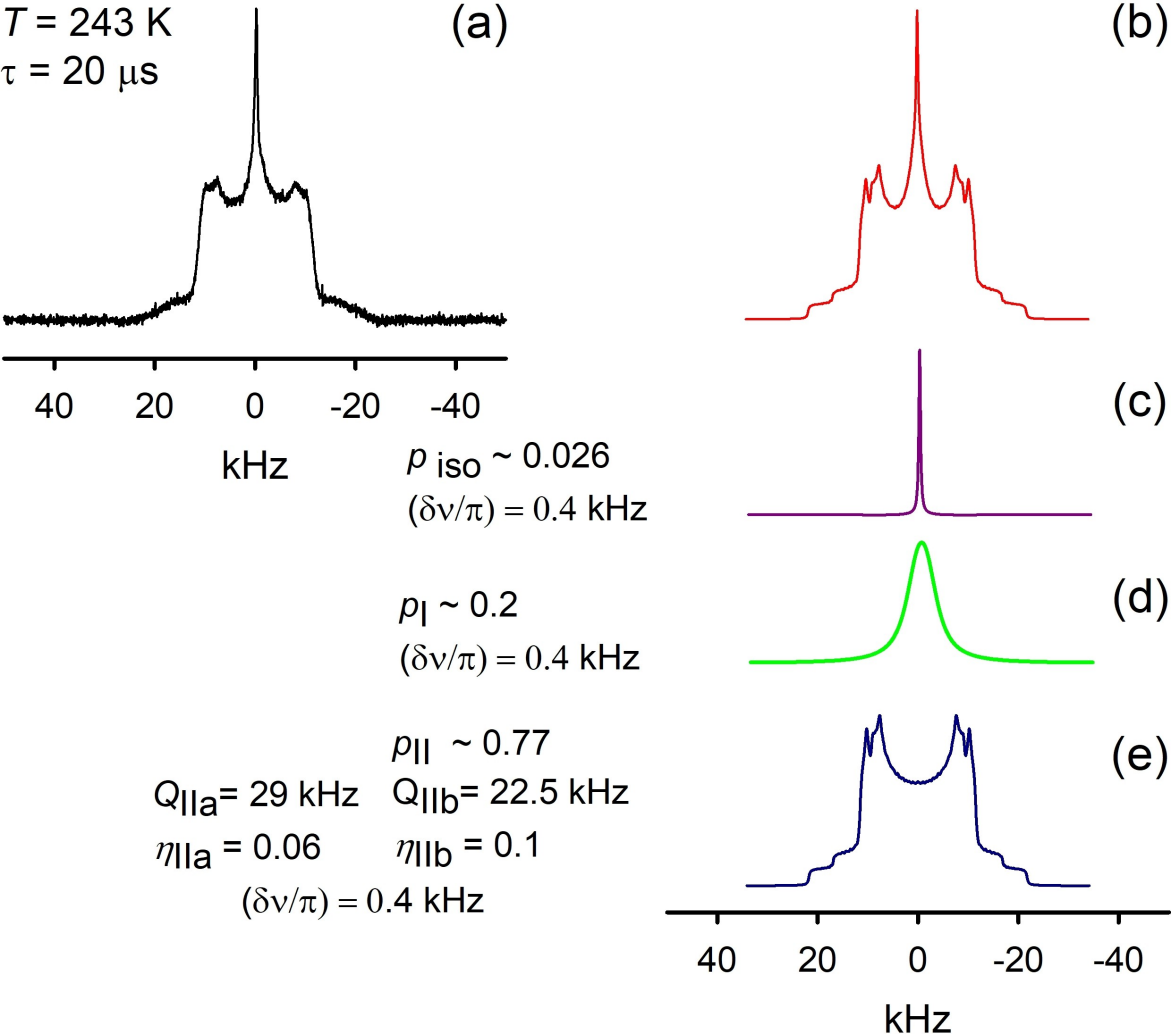
^2^H NMR spectra deconvolution of [C_8_H_17_PD_3_][NTf_2_] at *T*=243 K (a, b) shows the presence of the remaining fraction of d) the low temperature phase *I*_t_ , along with c) and e) the new phase components.

**Figure 12:**
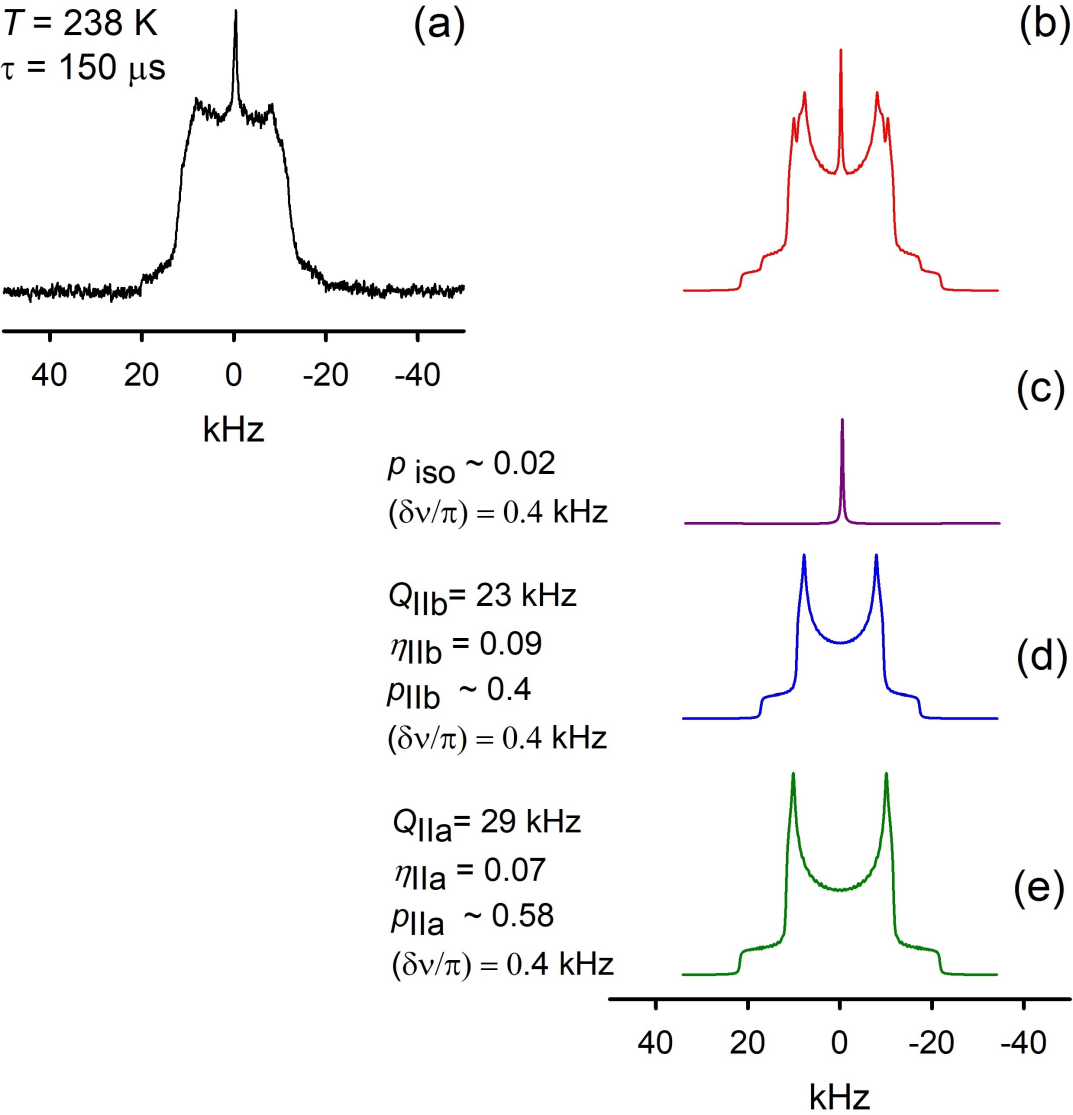
^2^H NMR spectra of [C_8_H_17_PD_3_][NTf_2_] measured with *T*_2_‐filtration deconvolution at *T*=238 K: a) experimental, b) simulated, c), d), e) deconvoluted spectra .

In the new phase the anisotropic patterns are now composed by two Pake‐powder components (Figure 11) with slightly different effective DQCC parameters *II*_a_ with (*Q*_IIa_=29 kHz, *η*_IIa_=0.07) and *II*_b_ with (*Q*_IIb_=23 kHz, *η*_IIb_=0.09). As expected for a supercooled phase, it shows the dynamical heterogeneity, as in addition to the anisotropic component an ultra‐narrow isotropic signal is present in the central part of the spectrum. This appears to be a common phenomenon for all solidified PILs.[^18^, ^19^] Upon further heating, the anisotropic component fully disappears above 263 K which indicates a full dynamical melting of the PIL. At the same time, the experimental patterns suggest that the low temperature phase *I*_t_ does not disappear in a step‐like manner and co‐exists within the new phase at least up to 253 K (Figures 12 and S2).

Hence, we can regard the whole 223–263 K region as a transitional one. The spectral deconvolution allows deriving populations for each of the phase components and providing unique information on the thermodynamics of dynamically heterogeneous super cooled phase. In the van't Hoff equation [Eq. (1)] the equilibrium constant is computed as the ration between relative populations of the anisotropic state *II* and the mobile state iso, i. e., $K_{eq}=p_{iso}/p_{II},$ as a function of the temperature (see Figure 6(c) and (d). The van‘t Hoff plot yields the molar enthalpy change *ΔH* ^Θ^ for the transition from heterogeneous to fully isotropic mobile state (Δ*H* ^*Θ*^=61 kJ mol^−1^, Δ*S* ^*Θ*^=223 J mol^−1^ K^−1^), suggesting the complete disappearance of hydrogen bonding.

At this stage we should discuss the dynamics in the transition phase. The increasing librations in the low temperature phase *I*_t_ indicate that before the transition actually starts, the still homogeneous phase gradually becomes dynamically disordered. On the other hand, the abrupt change of both *T*_1_ and *T*_2_ in the transient state (step‐change between 238 K and 243 K) is typical manifestation of a phase transition, which is surprisingly well above in temperature to the DSC results. The presence of two signals points out that in the new supercooled phase, there exists two types of hydrogen bonded C_8_H_17_PD_3_ cations. Both are involved into fast hydrogen bond rearrangement via axial rotation. The clear presence of the asymmetry additionally indicates that their respective PD_3_ fragments are capable to librate in an anisotropic fashion. Despite such motion of hydrogen bonds in PIL was recently reported and characterized,^[19]^ the present result is the first observation of a PIL offering a slightly different hydrogen bonding environment (which is reflected in the different DQCCs and asymmetry parameters).

Notably, this is fully consistent with the observations made in the low temperature phase, where the two types of PD_3_ environments were observed. The changes in the DQCC indicates, that while one of the species bonding remained almost the same, the second one underwent rearrangements to form a proper hydrogen bond strong enough to affect the quadrupolar interaction.

The relaxation times of the anisotropic components show a similar behavior to the low temperature phase, with just a slightly different rate constants of the motions, hence the hydrogen bonding potential is not strongly altered upon the phase transition. On the other hand, the relaxation of the isotropic component shows somewhat chaotic behavior within the transition region, which is expected for a highly heterogeneous state.

The cation mobility in the isotropic state can be further analyzed in detail in the already homogeneous dynamically melted state manifested above 263 K (Figure S3) by following the spin‐relaxation curve of the central signal (Figure 7(c). The two curves show very peculiar behavior: the spin‐lattice relaxation time temperature dependence curve shows a monotonous growth with a clear and strong increase of the curve slope around *T*∼303 K. This indicates the presence of two types of motion with notably different activation barriers. At the same time the transverse relaxation curve shows a growth behavior specific for a single type fast motion. Since in the upper temperature limit (303–343 K) the two curves are parallel, we can conclude that the *T*_2_ relaxation and the *T*_1_ high temperature limit are governed by a motion of the same nature. Since the *T*_2_ relaxation has the same slope in both low (263–303 K) and high temperature regions, we must conclude that the curve reflects the isotropic diffusion of the cation (obviously the C‐PD_3_ fragment is not capable to rotate in an isotropic fashion by itself). This also means, the lower barrier motion that governs the *T*_1_ at lower temperatures has to be an anisotropic rotation, which is expected considering the line shape analysis in the solid phase.

When the isotropic motion is present, the theoretical description of the spin relaxation becomes less complicated: as the quadrupole coupling tensor, in general anisotropic, is traceless by nature.^[9a]^ Thus, the apparent coupling tensor collapses to zero in the presence of a sufficiently fast (*τ*_c_ ≪ *Q*_0_^−1^) isotropic motion.

In the present case, we have first assumed the simplest model, including the anisotropic uniaxial rotation in a cone ${k_{C3}}^{iso}$ of the ‐PD_3_ fragment and the isotropic rotation with rate ${k_{D}}^{iso}$ , where *iso* stand for the isotropic spectrum component. Unfortunately, such a simple model does not fit well both *T*_1_ and *T*_2_ curve at once: the isotropic ${k_{D}}^{iso}$ (${E_{D}}^{iso}$ =41.5 kJ mol^−1^; $\frac{{k_{D0}}^{iso}}{2\pi }$ =2.5×10^14^) perfectly describe the *T*_2_ curve, the ‐PD_3_ rotation ${k_{C3}}^{iso}$ (${E_{C3}}^{iso}$ =12 kJ mol^−1^; $\frac{{k_{C30}}^{iso}}{2\pi }$ =7×10^11^) fits well the low temperature region of the *T*_1_ curve. Notably, the kinetic parameters for the internal 3‐site rotations in the liquid state are quite similar to the same rotations in the glassy state(s). The rotation geometry (the semi‐cone angle) *θ*_C3_^iso^=68° is quite close to the expected C‐PD_3_ rotation angle ∼69.5° and thus correlates well with the line shape analysis results. Yet, the high temperature region of the *T*_1_ curve is not reproduced well. This means, that there is an additional, anisotropic motion. Technically, we can add one in the similar manner as it was done for the anisotropic relaxation case. Numerical fitting allows to derive the nature of this additional motion: this new anisotropic motion affects primarily the *T*_1_ relaxation and thus is fast. For the C‐PD_3_ fragment there are only two options: low‐angle restricted symmetric librations of C−P bond or an anisotropic rotation. Simulations trials have shown that the best description of the experimental curves is provided when the second motion is described as a 3‐site uniaxial rotation with ${{\tilde{k}}_{C3}}^{iso}$ (${{\tilde{E}}_{C3}}^{iso}$ =41.5 kJ mol^−1^; $\frac{{{\tilde{k}}_{C30}}^{iso}}{2\pi }$ =8.5×10^14^). Notably, the rotation angle *θ*_2C3_^iso^=62°–64°, is also quite close to the geometry of the internal rotation of the ‐PD_3_ fragment, while the activation barrier and rates are similar to the isotropic rotation ${k_{D}}^{iso}\;$ kinetic parameters. This can be rationalized in the following manner: in general, the cation is hydrogen bonded with the anion with its PD_3_ fragment rapidly rotating about the 3‐fold axis. It is also able to rotate in an isotropic fashion with a much higher barrier, which probably means that the isotropic rotation is an individual process for the cation and not the whole ion pair. Thus, the cation must get detached from the anion with its ‐PD_3_ fragment. Hence, there exists a parallel process when the ‐PD_3_ detaches but instead of a full isotropic turnover is trapped back to (potentially) another cation with rate ${{\tilde{k}}_{C3}}^{iso}$ . This essentially anisotropic motion is close in its geometry to the internal uniaxial rotation as the re‐attachment occurs with randomized positions of the ‐PD_3_ deuterons. Hence, in addition to the full isotropic tumbling, the ‐PD_3_ fragment has effectively internal uniaxial rotations happening during the detachment/re‐attachment process.

To test this model, we have additionally measured the same sample at a different ^2^H NMR Zeeman frequency (at 38.38 MHz). The numerical fits of the experimental curve (Figure 7(d) show that our model describes these additional results well and hence is valid. This means that we are able to provide strong indications that in the [C_8_H_17_PD_3_][NTf_2_] PIL the cations dynamics and rotational diffusion is notably decoupled from the anion.

## Conclusion

In this study, we report the experimental characterization of the microscopic rearrangement within the ionic pair a PIL in the solid, super cooled and liquid states. We show the first example of the tunneling‐driven high (>100 K) temperature dynamics of hydrogen in a solid PIL. We provide direct ^2^H NMR experimental evidence of a complex phase behavior in this glass‐forming PIL, which would be rather non‐accessible by other methods, primarily by the DSC. We show that combined line shape and spin relaxation allow to map and correlate the phase and dynamical changes within the PIL in the broad temperature range from 71 K to 343 K. Finally, we show that in the super cooled state of the liquid phase there exists two states of hydrogen bonds with slightly different dynamics, while in the glassy phase only one type of hydrogen bond exists. To our knowledge, this is the first direct evidence of such microscopic rearrangement in a PIL. We also analyzed the enthalpy and entropy changes for the phase transitions.

## Experimental Section

We synthesized the deuterated PIL d3‐octyl phosphonium bis(trifluoromethylsulfonyl)imide [C_8_H_17_ **PD_3_**][NTf_2_] following the protocols as described in the Supporting Information. First, we synthesized the deuterated precursor materials d2‐octylphospine (base) and d1‐bis(trifluoromethanesulfonyl)imide (acid). Both compounds then reacted in stochiometric amounts in a glove box to yield the target colorless ionic liquid d3‐octyl phosphonium bis(trifluoromethylsulfonyl)imide.

The DSC thermogram (Figure 1) reveals that, upon cooling, the PIL first passes to a supercooled state and glassifies at about *T*_g_∼206 K. Remarkably, at further cooling there appears two additional transitions at about *T*_s1_∼186 K and *T*_s2_∼158 K, respectively. These transitions suggest the presence of some additional solid‐solid phase transitions at temperatures below *T*_g_. In order to cover the whole DSC traces, we have performed solid state ^2^H NMR measurements on the PIL with selectively deuterated hydrogen bonds for a broad temperature range *T*=67–333 K.

The ^2^H solid‐state NMR experiments above 100 K were performed at Larmor frequency $\omega_{z}/2\pi$ =61.42 MHz on a Bruker Avance‐400 spectrometer, using a high‐power probe with 5 mm horizontal solenoid coil. The ^2^H spectral patterns below 100 K were measured at Larmor frequency $\omega_{z}/2\pi$ =61.42 MHz on a Bruker Avance II+ 400 spectrometer, using a helium‐cooled high‐power probe with 5 mm horizontal solenoid coil. All ^2^H NMR spectra were obtained by Fourier transformation of quadrature‐detected phase‐cycled quadrupole echo arising in the pulse sequence (90°_*x*_–*τ*_1_–90°_*y*_–*τ*_2_–acquisition – *t*), where *τ*_1_=20 μs, *τ*_2_=21 μs and *t* is a repetition time of the sequence during the accumulation of the NMR signal. The duration of the π/2 pulse was 1.2–2.1 μs. Spectra were typically obtained with 50–20000 scans with repetition time ranging from 1 to 30 seconds.

## Conflict of interest

The authors declare no conflict of interest.

1

## Supporting information

As a service to our authors and readers, this journal provides supporting information supplied by the authors. Such materials are peer reviewed and may be re‐organized for online delivery, but are not copy‐edited or typeset. Technical support issues arising from supporting information (other than missing files) should be addressed to the authors.

Supporting Information

## Data Availability

The data that support the findings of this study are available from the corresponding author upon reasonable request.
