# Supplementary material for: High‐Temperature Quantum Tunneling and Hydrogen Bonding Rearrangements Characterize the Solid‐Solid Phase Transitions in a Phosphonium‐Based Protic Ionic Liquid
Source: Chemistry. 2022 Mar 28;28(23):e202200257. doi: 10.1002/chem.202200257 (PMC9311734; doi:10.1002/chem.202200257)
Supplement: Supplementary file 1 — Supporting Information [file CHEM-28-0-s001.pdf]

Chemistry–A European Journal

Supporting Information

High-Temperature Quantum Tunneling and Hydrogen Bonding Rearrangements Characterize the Solid-Solid Phase Transitions in a Phosphonium-Based Protic Ionic Liquid

Alexander E. Khudozhitkov, Masaki Donoshita, Alexander G. Stepanov, Frederik Philippi, Daniel Rauber, Rolf Hempelmann, Hiroshi Kitagawa, Daniil I. Kolokolov,* and Ralf Ludwig*

Supporting Information

1. **Synthesis and Characterization**
2. **Solid state NMR spectroscopy**

1. Synthesis and characterization

Diethyl octylphosphonate

100 mL triethyl phosphite (96 g / 578 mmol / 1.00 eq) and 110 mL 1-bromooctane (122 g / 632 mmol / 1.09 eq) were heated together to 160°C for two days. The ethyl bromide formed in the course of the reaction was distilled off through a Vigreux column. Distillation of the resulting crude product at 95°C at approximately 0.1 mbar yielded 85.9 g octylphosphonic acid diethyl ester (343 mmol / 59% yield).

^{1}H NMR ($CDCl_3$, 400 MHz, δ in ppm): 4.10-3.90 (m, 4H, P-O-***CH_2***-CH_3), 1.72-1.60 (m, 2H, P-***CH_2***-$(CH_2)_7$-H), 1.59-1.44 (m, 2H, P-CH_2-***CH_2***-$(CH_2)_6$-H), 1.38-1.11 (m, 10(+6)H, P-$(CH_2)_2$-***$(CH_2)_5$***-CH_3), 1.26 (t, $^{3}J_{H/H}$ = 7.1 Hz, (6H), P-O-CH_2-***CH_3***), 0.81 (t, $^{3}J_{H/H}$ = 6.8 Hz, 3H, P-$(CH_2)_7$-***CH_3***).

$^{13}C\{^{1}H\}$ NMR ($CDCl_3$, 101 MHz, δ in ppm): 61.35 (d, $^{2}J_{C/P}$ = 6.3 Hz, P-O-***CH_2***-CH_3), 31.82 (s, P-$(CH_2)_5$-***CH_2***-$(CH_2)_2$-H), 30.64 (d, $^{3}J_{C/P}$ = 16.9 Hz, P-$(CH_2)_2$-***CH_2***-$(CH_2)_5$-H), 29.07 (s, P-$(CH_2)_3$-***$(CH_2)_2$***-$(CH_2)_3$-H), 25.73 (d, $^{1}J_{C/P}$ = 140.4 Hz, P-***CH_2***-$(CH_2)_7$-H), 22.65 (s, P-$(CH_2)_6$-***CH_2***-CH_3), 22.43 (d, $^{2}J_{C/P}$ = 5.2 Hz, P-CH_2-***CH_2***-$(CH_2)_6$-H), 16.50 (d, $^{3}J_{C/P}$ = 6.0 Hz, P-O-CH_2-***CH_3***), 14.09 (s, P-$(CH_2)_7$-***CH_3***).

$^{31}P\{^{1}H\}$ NMR ($CDCl_3$, 162 MHz, δ in ppm): 32.56 (s).

d2-octylphosphine

2.5 g lithium aluminum deuteride (59.5 mmol / 1.30 eq.) were weighed in a glovebox and transferred under argon atmosphere into a three-necked round bottom flask equipped with reflux condenser, dropping funnel and conical ground joint stopcock. After 90 mL of dry diethyl ether were added to the lithium aluminum deuteride, the flask was cooled to 0°C in an ice bath. Subsequently, 12.0 mL diethyl octylphosphonate (11.4 g / 45.8 mmol / 1.00 eq.) in 50 mL of dry diethyl ether were added dropwise over 1 h. After addition was complete, the mixture was slowly warmed to room temperature while stirring for 14 h. The flask was then cooled to 0°C again and the solution quenched by the dropwise addition of 20 mL degassed NaOD (20%) solution in D_2O. After allowing to heat to ambient temperature with stirring for 4 h, the ethereal layer was transferred into a Schlenk flask by means of a cannula and the aqueous solution extracted with another portion of diethyl ether. The organic solvent was distilled off the combined ethereal phases, and the resulting residue was distilled at 35-45°C and approximately 2.3 mbar. 5.90 g of the product d2-octylphoshine (39.8 mmol / 87% yield) were obtained as a colorless liquid.

^{1}H NMR ($CDCl_3$, 400 MHz, δ in ppm): 1.57-1.42 (m, 4H, P-***(CH₂)₂***-(CH₂)₅-CH₃), (m, 10H, P-(CH₂)₂-***(CH₂)₅***-CH₃), 0.88 (t, $^{3}J_{H/H}$ = 7.1 Hz, 3H, P-(CH₂)₇-***CH₃***).

$^{13}C\{^{1}H\}$ NMR ($CDCl_3$, 101 MHz, δ in ppm): 32.99 (d, $^{2}J_{C/P}$ = 3.2 Hz, P-CH_2-***CH_2***-$(CH_2)_6$-H), 32.01 (s, P-$(CH_2)_5$-***CH_2***-$(CH_2)_2$-H), 30.72 (d, $^{3}J_{C/P}$ = 5.8 Hz, P-$(CH_2)_2$-***CH_2***-$(CH_2)_5$-H), 29.35 (s), 29.34 (s), 22.81 (s, P-$(CH_2)_6$-***CH_2***-CH_3), 14.24 (s, P-$(CH_2)_7$-***CH_3***), 13.66 (d, $^{1}J_{C/P}$ = 7.0 Hz, P-***CH_2***-$(CH_2)_7$-H).

$^{31}P\{^{1}H\}$ NMR ($CDCl_3$, 162 MHz, δ in ppm): −139.93 (p, $^{1}J_{P/D}$ = 30.62 Hz).

^{31}P NMR ($CDCl_3$, 162 MHz, δ in ppm): −137.95 (pt, $^{1}J_{P/D}$ = 30.59 Hz, $^{2}J_{P/H}$ = 6.3 Hz).

d1-bis(trifluoromethanesulfonyl)imide

6.76 g of lithium bis(trifluoromethanesulfonyl)imide (23.5 mmol / 1.00 eq.) and 5 mL of 98% d2-sulfuric acid (9.15 g / 93.3 mmol/ 3.96 eq.) were placed in a sublimation apparatus filled with argon at ambient pressure and heated to 100°C for 7 hours with gentle stirring. After cooling to ambient temperature, the apparatus was transferred into a glove box and the deposited product collected. The deuterated title compound was obtained as a white solid (3.28 g / 11.7 mmol / 49% yield) and further stored in the glove box.

$^{19}F\{^{1}H\}$ NMR ($CDCl_3$, 376 MHz, δ in ppm): −75.00 (s).

d3-octylphosphonium bis(trifluoromethanesulfonyl)imide

3.06 g of d1 bis(trifluoromethanesulfonyl)imide (10.9 mmol / 1.00 eq.) were weighed into a glass vial in a glove box and 1.60 g d2-octylphosphine (10.9 mmol / 1.00 eq) were added. The glass vial was sealed with a Teflon cap and allowed to react with occasional shaking for one day. After this time a magnetic stir bar was added and the liquid stirred for 2 hours. 4.66 g of the threefold deuterated product (10.9 mmol /quantitative yield) were obtained as colorless liquid.

^{1}H NMR (D_3CCN, 400 MHz, δ in ppm): 2.31 (dt, $^{2}J_{H/P}$ = 16.0 Hz, $^{3}J_{H/H}$ = 7.7 Hz, 2H, P-***CH_2***), 1.79-1.65 (m, 2H, P-CH_2-***CH_2***), 1.51-1.49 (m, 2H, P-$(CH_2)_2$-***CH_2***), 1.40-1.24z (m, 8H, P-$(CH_2)_3$-***$(CH_2)_4$***), 0.92 (t, $^{3}J_{H/H}$ = 6.6 Hz, 3H, P-$(CH_2)_7$-***CH_3***).

^{2}H NMR (H_3CCN, 61 MHz, δ in ppm): 1.91 (s).

$^{13}C\{^1H\}$ NMR (D_3CCN, 101 MHz, δ in ppm): 120.85 (q, $^1J_{C/F}$ = 320.6 Hz, ***CF_3***), 32.35 (s), 30.32 (d, $^3J_{C/P}$ = 14.9 Hz, P-$(CH_2)_2$-***CH_2***), 29.50 (s), 29.28 (s), 24.97 (d, $^2J_{C/P}$ = 4.9 Hz, P-CH_2-***CH_2***), 23.24 (s), 14.27 (s, P-$(CH_2)_7$-***CH_3***), 11.36 (d, $^1J_{C/P}$ = 48.2 Hz, P-***CH_2***-$(CH_2)_7$-H)

$^{31}P\{^1H\}$ NMR (D_3CCN, 162 MHz, δ in ppm): −54.07 (s).

^{31}P NMR (D_3CCN, 162 MHz, δ in ppm): −54.00 (br).

$^{19}F\{^1H\}$ NMR ($CDCl_3$, 376 MHz, δ in ppm): −80.12 (s).

2. Solid state NMR spectroscopy

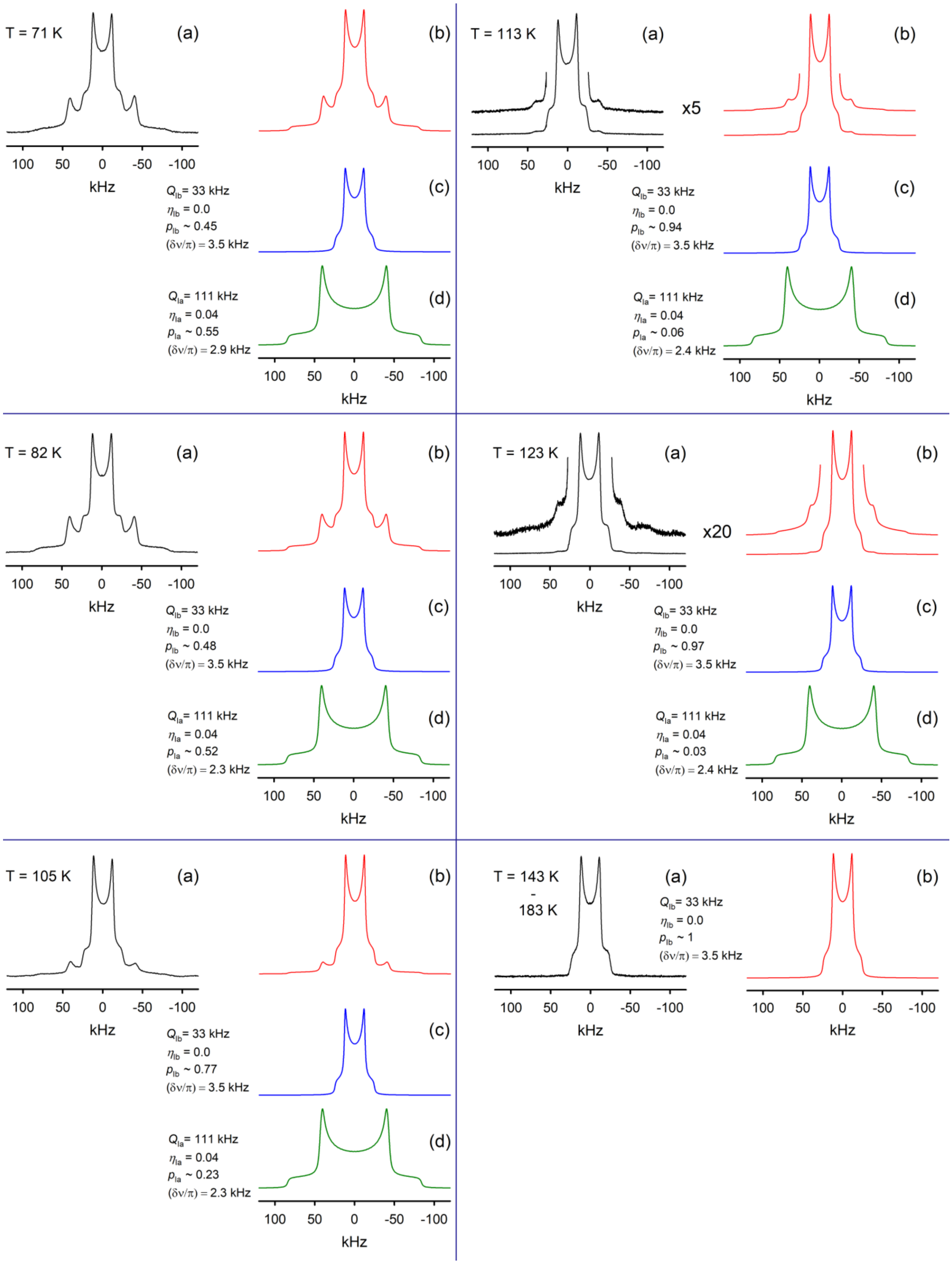

Figure S1. ^{2}H NMR spectra deconvolution for $[C_8H_{17}PD_3][NTf_2]$ at temperatures between 71 K and 183 K: (a) – experimental; (b) – simulated; (c) ***It*** component; (d) ***Is*** component.

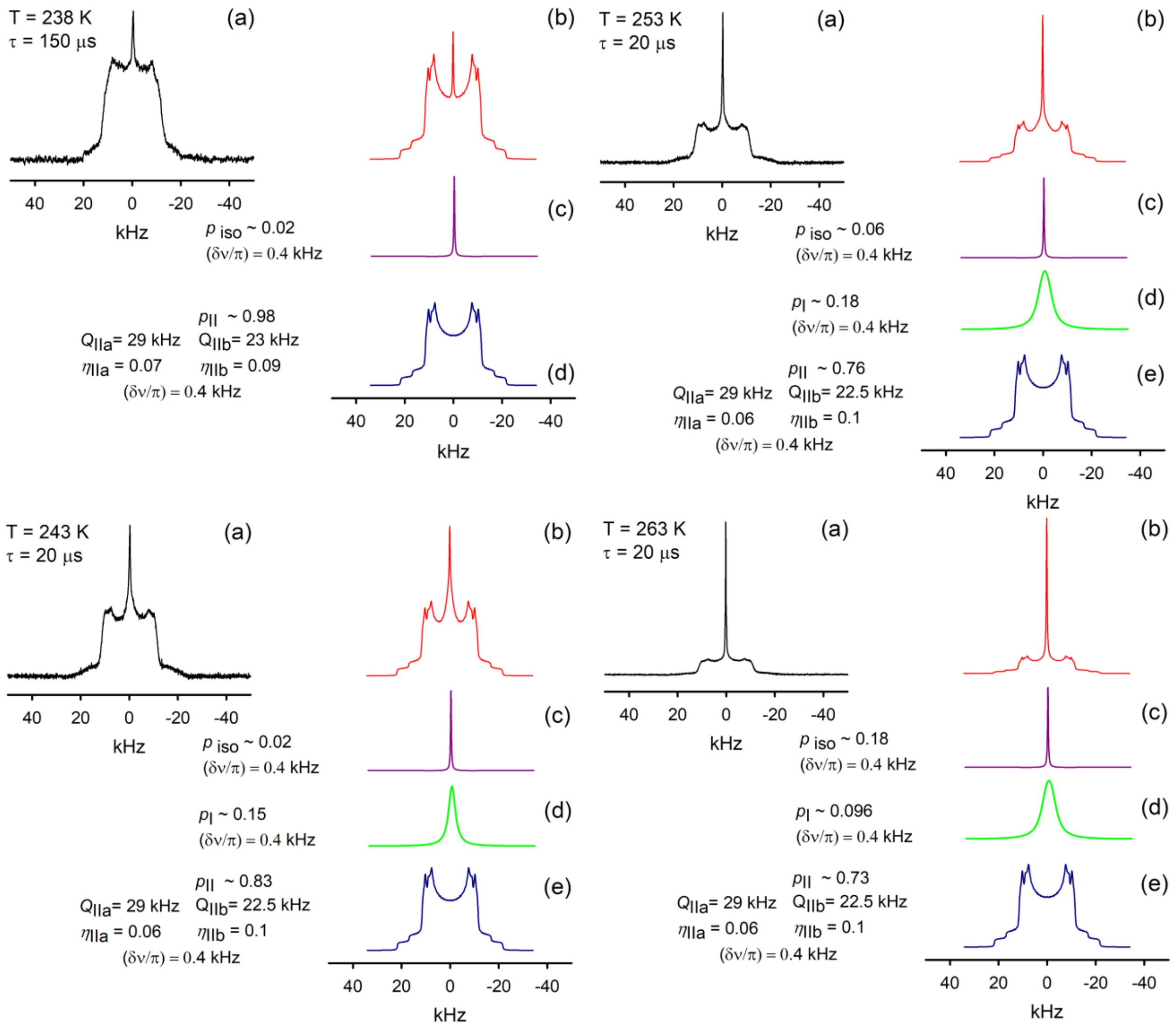

Figure S2. ^{2}H NMR spectra deconvolution for $[C_8H_{17}PD_3][NTf_2]$ at temperatures between 238 K and 263 K: (a) – experimental; (b) – simulated; (c) $\boldsymbol{I_{iso}}$ component; (d) ***I*** component; (e) combined ***II*** component.

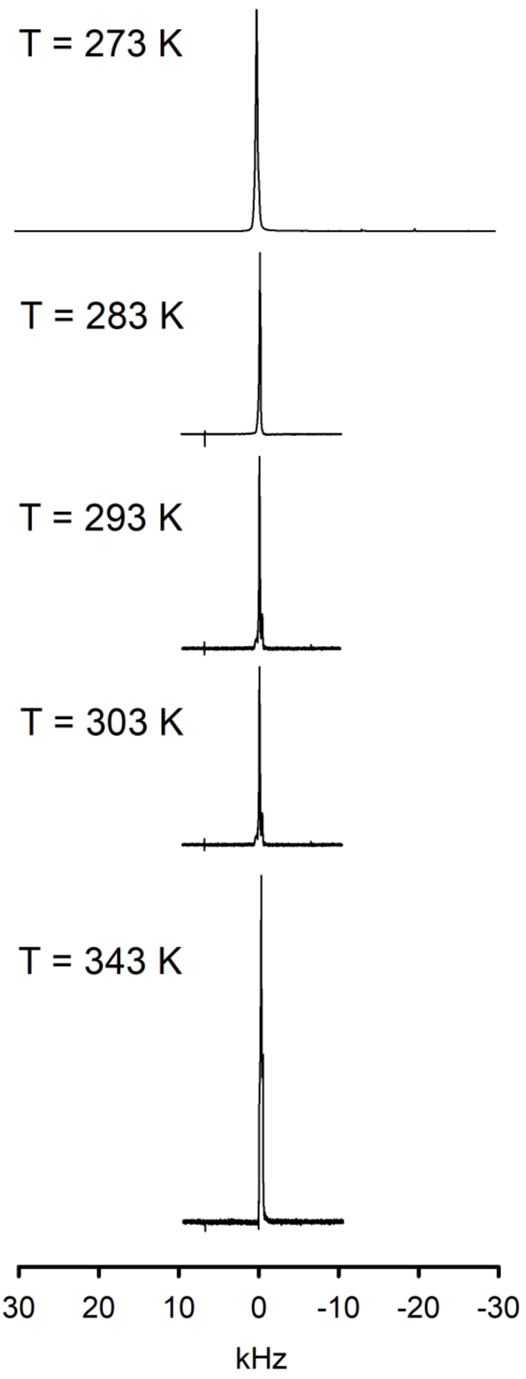

Figure S3. ^2H NMR spectra $[C_8H_{17}PD_3][NTf_2]$ at temperatures between 273 K and 343 K.
